# Knowledge and Awareness of Syncope Among the Population of Riyadh: A Cross-Sectional Study

**DOI:** 10.7759/cureus.28499

**Published:** 2022-08-28

**Authors:** Mohannad A Alghamdi, Faisal A Alshahrani, Faisal A Aldihan, Nawaf M Alamer, Fahad A Al Dihan, Aamir Omair, Ihab Suliman, Mohamud Mohamud

**Affiliations:** 1 College of Medicine, King Saud Bin Abdulaziz University for Health Sciences, Riyadh, SAU; 2 Medical Education, King Saud Bin Abdulaziz University for Health Sciences, Riyadh, SAU; 3 Cardiology, Cardiac Center, King Abdulaziz Medical City, Riyadh, SAU; 4 Epidemiology, Somali Center for Public Health, London, GBR

**Keywords:** cardiogenic, orthostatic, vasovagal, loss of consciousness, awareness, syncope

## Abstract

Background

Although syncope is a common emergency in medical settings, no research has yet evaluated the general population’s awareness regarding it. This study investigated the general population’s knowledge and awareness of syncope and if they could differentiate syncopal and non-syncopal causes of transient loss of consciousness (TLOC).

Methodology

A cross-sectional study was conducted in Riyadh through a validated, self-administered Arabic questionnaire that was distributed to the general population through social media using Google Forms (convenience sampling). Participants younger than 18 or not from Riyadh were excluded from the study. Two cardiologists validated the questionnaire, following which forward and backward translation was done. The questionnaire contained three sections. The first section included demographic data and chronic conditions. In the second section, participants were asked if they or one of their relatives had ever experienced syncope. The third section had eight scenarios assessing the participants’ syncope knowledge. Subjects with ≥five correct answers were considered to be aware.

Results

The number of total responses was 405 participants. Regarding demographic data, 53% of the participants were female, 33% had a medical background, and 76% had a university degree (n = 214, n = 134, and n = 306, respectively). The mean age of the participants was 33.2 ± 13.3 years. Participants who were aware of syncope represented 55% (n = 221). Among the syncope cases, orthostatic syncope had the highest number of correct answers (79%, n = 319), followed by vasovagal syncope (61%, n = 246). Males performed better in cases one (p = 0.001), two (p = 0.004), and seven (p = 0.01).

Conclusions

The results of this study showed that most participants were considered aware of syncope. Gender, marital status, and having a medical background had a significant influence on the results.

## Introduction

“Syncope is the sudden loss of consciousness, associated with the inability to maintain postural tone, with immediate and spontaneous recovery without requiring electrical or chemical cardioversion” [1]. The main cause of unconsciousness is primarily inadequate cerebral nutrients and oxygen delivery [2]. The prevalence of syncope for a lifetime of 70 years is 42%, with an annual rate of 6%. Moreover, the incidence ranges from 18.1 to 39.7 per 1,000 patients [1]. Further, syncope is a common medical emergency; in fact, the overall admission rate of syncope is 32%, which is 2% of all emergency department admissions [3]. In addition, syncope can affect the quality of life through the impairment of daily activities, which was measured to be 33% [4]. Most cases between the ages of 10 and 30 years were caused by vasovagal syncope (VVS) [5]. In addition, the incidence of syncope increases with age, especially after the age of 70 years, when it increases sharply [6]. Regarding the prevalence of syncope in association with gender, it was found that females had a higher prevalence rate [7].

Syncope is divided into reflex, orthostatic, and cardiac syncope. VVS, a type of reflex syncope, is the most common cause of syncope. Second, orthostatic syncope occurs when a patient’s blood pressure drops suddenly after standing up, leading to syncope. Finally, cardiac syncope is induced by cardiac rhythm or structural abnormality [8]. Although there are other causes of syncope, such as neurological causes, psychiatric disorders, and endocrinological causes, these types are less common [1]. Although the different types of syncope have different triggers, they share the exact pathophysiology. It starts with a decreased venous return to the heart. Consequently, mechanoreceptors in the left ventricle will send signals to the central nervous system. Ultimately, the signals would cause an increase in the parasympathetic tone and a decrease in the sympathetic tone leading to loss of consciousness [9].

Even though syncope is the most encountered emergency in the dental setting, most dentists were not confident enough to manage it [10-12]. One research conducted in Croatia showed that 84% of dental emergencies were VVS. Also, 46% of the General Dentists (GD) were not competent in dealing with syncope. Moreover, the more training the GD underwent, the more confident they were in dealing with emergencies, except for VVS [10]. A similar local study in Saudi Arabia from Qassim showed syncope was the most commonly reported dental emergency, accounting for 28% of all cases [11]. Another study conducted in the Eastern Province showed that VVS is the most common emergency at 53%. Moreover, one-third of the participants did not know how to manage medical emergencies or were not competent to deal with them [12].

Most global and local literature focuses on assessing the management of medical emergencies among targeted groups, especially dentists and health caregivers, but not the general population. Furthermore, the focus was not on syncope and included other medical emergencies, such as cardiac arrest and anaphylaxis. Besides, no research has evaluated the awareness of those who have comorbidities that predispose them to syncope. This study aimed to evaluate the general population’s knowledge and awareness of syncope and if they could differentiate between syncopal and non-syncopal causes of transient loss of consciousness (TLOC). In addition, the association between baseline characteristics (e.g., gender, level of education, and comorbidities) and the level of awareness of syncope was assessed.

## Materials and methods

This cross-sectional study targeted the general population of Riyadh to explore their knowledge and awareness about syncope. The participants were all 18 years and above, including both genders, and Saudis and non-Saudis living in Riyadh, Saudi Arabia. Google Forms were used to distribute the questionnaire through social networks, Facebook, and Twitter (convenience sampling). Riyadh’s population is almost 8.6 million according to the General Authority for Statistics [13]. Considering the margin of error of 5% and the confidence level of 95%, the required sample size was estimated to be 385. Calculations were done using Raosoft, and we assumed 50% prevalence as no previous studies could be taken as a reference [14]. The Institutional Review Board (IRB) of King Abdullah International Medical Research Center (KAIMRC), Riyadh, Saudi Arabia, granted ethical approval (SP20/381/R). At the beginning of the survey, a statement was provided for the participants to express their consent to be involved in the study. The study objectives were provided to the participants, and participation was voluntary.

Questionnaire development

An Arabic version of the survey was used in the research. Backward and forward translation methods were used in developing the survey. The questionnaire was first established in English; subsequently, it was given to a professional translator to be translated into Arabic. Backward translation was done from Arabic back to English using the same process mentioned before by giving it to a different professional translator. Lastly, both versions were compared. Furthermore, in the final version, simpler words were chosen to better fit the general population compared to the original version. The word “fainted” was changed to “loss of consciousness” in the final Arabic version to avoid misunderstandings that might occur in the Arabic language.

Content validity was achieved by giving the questionnaire to two cardiology consultants working at King Abdulaziz Cardiac Center in Riyadh. Then the questionnaire was edited according to their comments. Changes included removing some questions in the survey, which asked about smoking and what types of syncope the participants knew because both were not relevant to the study. Moreover, the definition of syncope in section three was removed as it was going to change the study’s goal from assessing participants’ knowledge and awareness to educating them and then assessing their understanding of the definition. In the diabetes case (second case), we changed the case from a patient losing his conciseness to only feeling dizzy to make it clearer. In the coma case (fifth case), the patient’s case changed from having a cardiac arrest to a coma to differentiate it from syncope.

The questionnaire included three sections. The first section consisted of demographic data such as age, gender, nationality, educational level, marital status, suffering from a chronic illness, and having a medical background. In the second section, participants were asked if they or one of their relatives had ever experienced syncope. The last section assessed the participants’ knowledge of syncope without providing a definition and features of syncope. There were eight case scenarios; three cases resembled a type of syncope, while the other five resembled other causes of loss of consciousness. The first, fifth, and seventh cases were about syncope (vasovagal, cardiogenic, and orthostatic, respectively), whereas the second, fourth, fifth, sixth, and eighth cases were about other causes of loss of consciousness (hypoglycemia, presyncope, coma, alcohol intoxication, and seizure, respectively). Participants had to answer and label each scenario as either an example of syncope or not. Participants who correctly labeled five or more out of the eight cases were considered aware of syncope, and those who labeled less than five cases were considered unaware of syncope. Further, the participants were not provided with a definition or any features of syncope to answer the scenarios.

Data analysis

For data entry and management, Microsoft Excel was used. For data analysis, SPSS version 22 (IBM Corp., Armonk, NY, USA) was used. Percentages and frequencies were presented for the qualitative variables (e.g., level of education, comorbidities, and gender), and mean and standard deviation were used to present the numerical variables (e.g., age). The chi-square test was used to test baseline characteristics’ association with the outcome variables. All tests were considered statistically significant if the p-value was <0.05.

## Results

In this study, 405 participants enrolled and completed the survey (Table 1). Regarding gender, the number of female participants was 214 (53%). The age of the participants ranged from 18 to 65 years, and the mean age was 33.25 ± 13.3 years. Out of the total number of participants, 95% were Saudis (n = 385). Most participants had a university degree (n = 306, 76%). Participants with a non-medical background accounted for 67% (n = 271) of the total sample. Regarding marital status, 53% were married (n = 213). Furthermore, participants who had experienced syncope in their lifetime were 36% of the total sample (n = 146), and 50% of the participant knew someone who had a syncope (n = 204). Most participants had no chronic diseases (n = 313, 79.8%). However, the most common chronic diseases were diabetes mellitus (n = 33, 8%), asthma (n = 22, 5%), and hypertension (n = 18, 4%) (chronic diseases are not represented in the tables).

**Table 1:** Participants’ characteristics (n = 405).

Baseline characteristics	n (%)
Gender	
Male	191 (47%)
Female	214 (53%)
Nationality	
Saudi	385 (95%)
Non-Saudi	20 (5%)
Education	
Up to high school	63 (16%)
University degree	306 (76%)
Postgraduate education	36 (9%)
Marital status	
Single	192 (47%)
Married	213 (53%)
Medical background	134 (33%)
Ever had syncope	146 (36%)
Know someone who had syncope	204 (50%)

**Figure 1:**
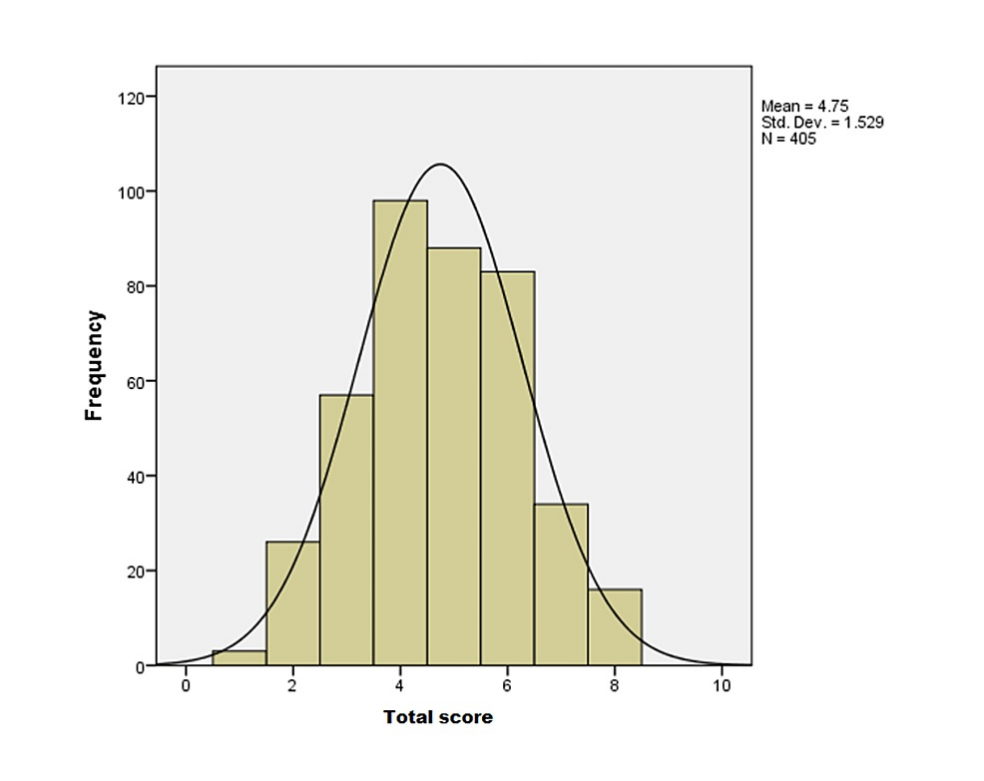
Frequencies of the total score of all participants.

The descriptive data presented in Table 2 show that scenario six had 335 (83%) correct answers, which was the highest of all scenarios. In addition, 319 (79%) and 324 (80%) participants correctly answered scenarios four and seven, respectively. In contrast, the fifth scenario (n = 252, 61%) and the eighth scenario (n = 239, 59%) had the lowest correct answers. Regarding the total score of the participants, the mean was 4.75 ± 1.52 (Figure 1). The study’s main finding demonstrated that 221 (54.6%) of subjects were aware of syncope (scored five or more). In addition, among the causes of syncopal loss of consciousness, orthostatic syncope had the highest number of correct answers (n = 319, 79%), followed by VVS (n = 246, 61%).

**Table 2:** Case scenarios. *: syncopal causes

Cases	Scenarios	Correct n (%)
C1^*^	A student in the primary school lost consciousness after she saw the needle for her common flu vaccination	246 (61%)
C2	A person with diabetes, who did not eat breakfast this morning, is presenting in front of a group. He felt dizzy and his heart was racing	175 (43%)
C3^*^	Your neighbor, who is suffering from cardiac disease, suddenly fell unconscious while praying. However, he gained consciousness immediately	204 (50%)
C4	A woman with anemia stood up suddenly and felt dizzy. She was going downstairs and was about to fall, but she held on to the handrails and managed to steady herself	324 (80%)
C5	After having severe injuries from a car accident, a patient was taken to the hospital and was unconscious until he finally woke 10 days later	153 (38%)
C6	A person under the influence of alcohol felt tipsy and fell	335 (83%)
C7^*^	An elderly man, who takes medication for his hypertension, was lying down. Suddenly, he stands up and then feels unsteady and falls	319 (79%)
C8	A person in a supermarket started to feel dizzy and after a while he fell unconscious. After the fall, he started to bite his tongue and having convulsions. After 10 minutes, he regained consciousness	166 (41%)

Table 3 demonstrates a difference between males and females; the former had significantly better scores in the first (p = 0.001), second (p = 0.004), and seventh (p = 0.01) scenarios. Regarding marital status, married people had better scores in the first (p = 0.018), second (p = 0.016), and seventh (p = 0.001) scenarios. However, single persons scored higher in the sixth scenario at p = 0.031. Moreover, people with a medical background generally performed better, especially at first (p = 0.02), second (p = 0.018), and sixth (p = 0.046) scenarios.

**Table 3:** Inferential statistics. *: significant p-value (≤0.05).

Cases	Gender		Education level			Marital status		Medical background		Nationality	
	Male	Female	Up to high school	University degree	Higher education	Single	Married	With	Without	Saudi	Non-Saudi
C1	69.1%, n = 132	53.3%, n = 114	55.6%, n = 35	63.4%, n = 194	47.2%, n = 17	54.7%, n = 105	66.2%, n = 141	71.6%, n = 96	55.4%, n = 150	60%, n = 231	75%, n = 15
	P = 0.001*		P = 0.112			P = 0.018*		P = 0.002*		P = 0.180	
C2	50.8%, n = 97	36.4%, n = 78	34.9%, n = 22	44.4%, n = 136	47.2%, n = 17	37%, n = 71	48.8%, n = 104	51.5%, n = 69	39.1%, n = 106	43.1%, n = 166	45%, n = 9
	P = 0.004*		P = 0.334			P = 0.016*		P = 0.018*		P = 0.868	
C3	50.8%, n = 97	50%, n = 107	57.1%, n = 36	48.4%, n = 148	55.6%, n = 20	52.6%, n = 101	48.4%, n = 103	52.2%, n = 70	49.4%, n = 134	49.9%, n = 192	60%, n = 12
	P = 0.875		P = 0.362			P = 0.393		P = 0.597		P = 0.377	
C4	77.5%, n = 148	82.2%, n = 176	82.5%, n = 52	79.7%, n = 244	77.8%, n = 28	81.3%, n = 156	78.9%, n = 168	78.4%, n = 105	80.8%, n = 219	80.5%, n = 310	70% n = 14
	P = 0.232		P = 0.828			P = 0.55		P = 0.561		P = 0.252	
C5	35.1%, n = 67	40.2%, n = 86	39.7%, n = 25	38.6%, n = 118	27.8%, n = 10	39.6%, n = 76	36.2%, n = 77	28.4%, n = 38	42.4%, n = 115	38.7%, n = 149	20%, n = 4
	P = 0.29		P = 0.426			P = 0.477		P = 0.006*		P = 0.093	
C6	82.7%, n = 158	82.7%, n = 177	84.1%, n = 53	81.7%, n = 250	88.9%, n = 32	87%, n = 167	78.9%, n = 168	88.1%, n = 118	80.1%, n = 217	82.3%, n = 317	90%, n = 18
	P = 0.997		P = 0.53			P = 0.031*		P = 0.046*		P = 0.377	
C7	84.3%, n = 161	73.8%, n = 158	82.5%, n = 52	78.4%, n = 240	75%, n = 27	71.9%, n = 138	85%, n = 181	79.1%, n = 106	78.6%, n = 213	78.4%, n = 302	85%, n = 17
	P = 0.01*		P = 0.65			P = 0.001*		P = 0.907		P = 0.484	
C8	38.7%, n = 74	43%, n = 92	54%, n = 34	38.6%, n = 118	38.9%, n = 14	42.2%, n = 81	39.9%, n = 85	38.1%, n = 51	42.4%, n = 115	41.6%, n = 160	30%, n = 6
	P = 0.386		P = 0.74			P = 0.641		P = 0.4		P = 0.305	

## Discussion

This study aimed to assess the general population’s knowledge and awareness regarding syncope and their ability to distinguish between syncope and other causes of loss of consciousness. The findings in our study suggested that more than half of the general population of Riyadh were aware of syncope, and medical background, gender, and marital status had the most significant influence on the results.

Even though local and international studies showed that syncope was the most common emergency encountered in the dental care setting, many medical caregivers were unable to manage it [10-12]. Similarly, our study’s participants with a medical background performed poorly in the fifth case, with only 28% correct answers. In the first case, married individuals performed better than single individuals; this might be because married participants have children who might have experienced VVS, especially in the 15-17 age group, where the incidence ranges from 28% up to 38% [15]. Our study found that participants with a medical background were better at identifying VVS (72%). Their academic advantage can explain this because they have encountered syncope in their practice. Further, a study conducted in the Netherlands showed that 39% of students had experienced syncope at least once [16].

Regarding VVS, male participants had better knowledge about VVS than female subjects, even though VVS is more common in the female population [17]. Although VVS is the most common type of syncope among the causes of syncopal loss of consciousness, orthostatic syncope had the highest number of correct answers, followed by VVS [8]. In the fifth (coma scenario) and eighth (seizure scenario) cases, both had the lowest percentage of correct answers, respectively. This could be explained by the low incidence rate of both coma (8.5 per 100,000 persons) and seizure (23-61 per 100,000 persons) compared to syncope (6.2 per 1,000 persons) [1,18,19].

One of the limitations we encountered in our study was language. This can be due to the misunderstanding of the word syncope and loss of consciousness in Arabic, unlike in English. One example is the coma scenario (case five) which had the lowest number of correct answers. Additionally, participants with a medical background performed poorly in this scenario. Another limitation was convenience sampling which could have altered the randomness and generalizability of our sampling to our population. Moreover, urban areas have higher education and medical background levels, and the study was conducted in an urban area, which could have altered the results of the study if rural parts were included. Moreover, because there were no other studies that evaluated the knowledge and awareness of syncope in the general population internationally or locally, we could not compare our findings with other studies. Lastly, our questionnaire was self-designed; therefore, there was no reference to previous questionnaires that we could have used.

Recommendations

Our research attempted to assess the extent of knowledge and awareness about syncope in the general population of Riyadh. Nevertheless, more research is needed in this area of study. Therefore, we emphasize that there should be more studies regarding syncope, in general, and more about knowledge and awareness, in particular. In addition, research should be conducted to evaluate medical personnel’s knowledge and awareness using an English-language questionnaire to exclude language limitations.

## Conclusions

This study aimed to assess the knowledge and awareness of syncope among the general population in Riyadh, Saudi Arabia. The main finding showed that more than half of the participants had more than five correct answers. Furthermore, married individuals and male participants performed better in the first, second, and seventh cases. The main predictors of awareness of syncope were gender, marital status, and having a medical background.
